# Influence of Group Identification on Malicious and Benign Envy: A Cross-Sectional Developmental Study

**DOI:** 10.3389/fpsyg.2021.663735

**Published:** 2021-06-30

**Authors:** Elena Gaviria, Laura Quintanilla, María José Navas

**Affiliations:** ^1^Department of Social and Organizational Psychology, National University of Distance Education, Madrid, Spain; ^2^Department of Methodology for Behavioral Sciences, National University of Distance Education, Madrid, Spain

**Keywords:** emotional development, benign envy, malicious envy, personal identity, social identity, group identification, schadenfreude

## Abstract

Envy is the result of a social comparison that shows us a negative image of ourselves. The present study addresses the effect of the context of group comparison and group identification on children's expression of this emotion. Through different stories, participants aged between 6 and 11 years were exposed to four contexts of upward social comparison in which they had to adopt the role of the disadvantaged character. From their emotional responses and their decisions in a resource allocation task, three response profiles were created: malicious envy, benign envy, and non-envy. Although we found important differences between verbal and behavioral responses, the results showed greater envy, both malicious and benign, when the envied was an out-group. On the other hand, when the envied belonged to the in-group and competed with a member of the out-group, malicious but not benign envy practically disappeared. With age, envious responses decreased, and non-envious responses increased. The role of social identity in the promotion and inhibition of envy is discussed, as well as the acquisition of emotional display rules in the benign envy and non-envy profiles.

## Introduction

Envy is an unpleasant social emotion that arises when we compare ourselves with others in terms of their characteristics and belongings and we perceive that they surpass us. This emotion of discomfort arises because the result of this upward comparison reveals our shortcomings. Envy is, therefore, a self-conscious emotion indicating a negative self-evaluation, or an inferior self-image with respect to others (Miceli and Castelfranchi, 2007; Smith and Kim, 2007; Van Dijk et al., 2015). There are two types of envy, benign and malicious (Van de Ven et al., 2009; Lange and Crusius, 2015). Benign envy does not imply that the envious individual harbors any ill wishes toward the envied individual, while malicious envy awakens the desire that the envied suffers some harm or misfortune and, if this occurs, the envious person rejoices (Van de Ven, 2016). This emotion, the joy caused by the misfortune of others, is termed *schadenfreude* in German. Since there is no term in either English or Spanish to name this emotion, this German term will be used hereon in. *Schadenfreude*, associated with malicious envy, is not always because the envied loses his/her advantage. It may occur because it can entail the opportunity to obtain that advantage lost by the other (Shamay-Tsoory et al., 2014).

The expression of envy and *schadenfreude* is considered socially undesirable, assuming a discomfort at something that benefits another person and rejoice over the misfortune he/she suffers. In fact, there are social rules on emotional expression (*display rules*), which have to do with knowing the appropriateness of showing certain emotional expressions in different social situations (Saarni, 1999).

### Children's Understanding of Envy and Schadenfreude

Studies have found a developmental pattern on understanding these emotions, between the ages of three and ten. From 3 to 5 years old, children attribute *schadenfreude* to an envious person when he/she witnesses the destruction of an object that the envied person possessed. However, from the age of six, they stop attributing *schadenfreude* and attribute pain or sadness (discomfort) at the destruction of the envied object. This response pattern has been explained appealing to the multifaceted nature of envy (Recio and Quintanilla, 2015; Jensen de López and Quintanilla, 2019), with some dimensions that are easier to understand than others. Thus, children from the age of three can understand which situations produce envy, even before verbally using the emotional label. That is, they understand the simplest dimension, which refers to the link between desires and their satisfaction: they realize that a person feels discomfort when she wants what another has and cannot obtain it, and they recognize the feeling of well-being of the owner of the desired object. From the age of six, the socio-moral dimension of envy begins to be understood, which also implies considering which emotions should be expressed or hidden according to display rules (Quintanilla and Giménez-Dasí, 2017; Quintanilla and Recio, 2019). This knowledge is part of the child's socialization, but it is also part of the development of interpersonal thinking. Since being envious implies recognizing a lack in oneself (being in an inferior position), and expressing it could be detrimental to one's reputation (a negative social evaluation), thus it is possible that children assume the rule of not showing envy to protect their public image (Quintanilla et al., 2018). Likewise, it is possible that they inhibit the attribution of *schadenfreude* considering that laughing at someone else's misfortune hurts the feelings of the other and is a socially reprehensible behavior (Nesdale, 2013).

Other studies have investigated the experience of *schadenfreude* and sympathy and their relationship with prosocial behavior (the attempt to help a person who suffers damage) in children aged between 4 and 8 years. Schulz et al. (2013) found that 8-year-old children modified their intention to help according to whether they had experienced *schadenfreude* or sympathy for someone who suffered harm. Steinbeis and Singer (2013) studied the effect of experiencing envy and *schadenfreude*, in children aged between 7 and 13, measured through resource allocation paradigms. Their results indicated that children choose egalitarian allocations when they experience less envy and *schadenfreude*. However, the decrease in these social emotions with age is only attributed to processes related to emotional regulation, but no reference is made to the role played by the context, in which the opponent with whom the resources are distributed is a member of another group.

There are two gaps that should be highlighted in envy studies with children. Firstly, as far as we know, the development of benign envy has not been studied. When participants do not attribute *schadenfreude* to the envious person, it is directly interpreted that they do not attribute envy (e.g., Jensen de López and Quintanilla, 2019). Secondly, no attention has been paid to the role of social comparison in group and intergroup settings. This study aims to contribute to correcting these deficiencies.

### The Group Context of Envy

In most of the research that has addressed children's understanding of envy the scenarios that are posed to the participants represent situations of interpersonal comparison. However, the group context meets all the antecedent conditions that are considered central -at least in research with adults- for envy to occur: the psychological similarity or closeness between the envied and the envious, social comparison, and the relevance of the dimension of comparison for the self-concept of the envious (Tesser, 1988, 1991; Van de Ven and Zeelenberg, 2020).

The feeling of being a member of a group implies perceiving oneself as similar and linked to the rest of the people who belong to that group (Tajfel, 1981; Tajfel and Turner, 1986). Similarity favors social comparison, since comparing ourselves with someone similar and close provides more relevant information for us than doing so with someone different (Festinger, 1954; Heider, 1958; Goethals and Darley, 1977); consequently, it has much more impact on our self-evaluation and our emotions (Smith, 2000). Similarity and psychological closeness can also foster envy through counterfactual thinking, which makes us think that it could have been us, and not the other, who obtained the advantage (Van de Ven and Zeelenberg, 2015). Moreover, the group can establish what is important and valued, and serve as a reference for comparison (Leach and Vliek, 2008). If another member has something that the group considers valuable and I, who also belong to the group, do not have it, it affects the recognition of my worth among my peers (Salice and Montes, 2018).

On the other hand, belonging to a group is part of our identity, which implies not only cognitive aspects, such as the perception of similarity, but also motivational, emotional, and behavioral aspects: attachment, consciousness of belonging to the group, positive evaluation of that belonging, empathy toward other members, and in-group favoritism (Tajfel, 1981; Turner et al., 1987; Smith et al., 1999; Cameron, 2004; Leach et al., 2008; Roth et al., 2019). Some of these processes could counteract the feeling of envy.

In the adult population, some studies have found a positive relationship between group identification (operationalized in terms of similarity) and envy (e.g., Schaubroeck and Lam, 2004; Duffy et al., 2012). In others, however, the results show a negative relationship (e.g., Brewer and Weber, 1994; McFarland and Buehler, 1995; Stapel and Koomen, 2001; Gardner et al., 2002; Chen and Li, 2009). Other studies suggest that group identification is only related to a certain type of envy. For example, Duffy et al. (2012) propose that, although group identification promotes envy due to the comparison with other similar, this same identification prevents envy from leading to feelings or actions that are harmful to the envied by strengthening the bonds between group members. In other words, only benign envy would develop (Van de Ven et al., 2009). The conclusion drawn from all these seemingly contradictory studies is that the key might lie in what kind of self (or what aspect of self-concept) is active at the time of comparison.

According to the self-categorization theory (Turner et al., 1987), when our personal self is active, we see ourselves as a unique person, and the differences with others are accentuated, due to a contrast effect. Thus, when comparing ourselves with others who surpass us, as these differences leave us in inferiority, our self-esteem suffers and we will probably feel envy or frustration. On the other hand, when it is our social self that is active, a depersonalization occurs, that is, we see ourselves as an interchangeable member within a social category, the similarities with the other are accentuated by an assimilation effect, and more value is attributed to being part of the same social unit. Therefore, when comparing ourselves with others, we are more likely to feel their superiority as a stimulus and inspiration (I can become like the other) or, as the social identity theory maintains, the superiority of other members of our group will make the group as a whole better and, thus, our social identity will be more positive, improving our self-esteem (Blanton et al., 2000).

The accentuation of the social self and the assimilation effect with the rest of the group members occur only in intergroup contexts. When the in-group constitutes the frame of reference for social comparison, the evaluation of the self is carried out in contrast to the attributes of other members of the group (Turner et al., 1987). According to this perspective, the in-group context would favor envy among the members of the group, while an intergroup setting would not be conducive to this type of feelings toward the members of the in-group. On the other hand, it would foster envy toward the out-group. If, when comparing my group with another, I perceive that my group is at a disadvantage, that will produce a negative social identity and I will feel envy toward the out-group. However, this will only happen if I feel identified with my group: the greater the identification, the greater the envy that I will feel toward a group that exceeds mine.

### Group Identification in Children

Various studies have found that from a fairly early age (between 3 and 5 years) children show preferences for their own group in the minimal group paradigm, although this favoritism is stronger at school age (e.g., Yee and Brown, 1992; Nesdale and Flesser, 2001; Nesdale et al., 2007; Dunham et al., 2011). This bias is evident when they have to make choices or distribute resources between their group and another, and also when they are asked to attribute positive or negative traits to their group and another group (Nesdale, 2001). Furthermore, from the age of five, children can subjectively identify with groups to which they belong and that are important to them, that is, they are able to include these groups in their self-concept (Bennett and Sani, 2008; Sani and Bennett, 2009).

According to the social identity development theory (SIDT, Nesdale, 2004), between the ages of six and eight, children begin to show a stronger preference for their in-group and differentiate it cognitively, affectively, and behaviorally from other groups. Also they consider the out-group members as similar among themselves, but different from in-group members (Nesdale and Flesser, 2001; Nesdale et al., 2004). At this age, attention is focused on the own group, and favoritism toward the in-group is not accompanied by hostility toward out-groups. These negative attitudes and behaviors toward out-groups appear later, when the focus of attention is broadened to include other groups and there are conditions that feed that hostility, such as group norms or a threat from the other group.

One implication of the studies that have documented the strong preference for the in-group at these ages is that what is important for children is to belong to a group and to be accepted by it. Therefore, any indication of rejection or threat to his/her group membership will have a negative impact on the child, and if the rejection is perceived as real, the effect will be greater both emotionally and behaviorally (Nesdale, 2008). However, to our knowledge, there are no studies that have addressed how children experience upward social comparison in a group or intergroup situation (e.g., if another member of the group is chosen in their place) when they identify with their own group.

The starting point of this study is the work of Recio and Quintanilla (2015) on the invidious comparison regarding tangible and intangible possessions in children. In one of the scenarios presented to the participants, there are two children from the same class who wish to be the representatives in a competition against children from another class. One of the two is chosen (the envied) to represent his/her group. When the day of the competition arrives, the chosen one suffers a mishap and loses the competition. The results of this research showed the response pattern mentioned previously: children, from 6 or 7 years of age, stop attributing *schadenfreude* to the envious when the envied suffers a mishap that eliminates his/her advantage. The interpretation was based on social desirability and the acquisition of emotional display rules.

This explanation would not pose any problem if it referred to an interpersonal comparison situation (Saarni, 1979; Nesdale, 2013). However, in the scenario described above, an alternative explanation could be considered taking a group rather than an individual perspective: It is possible that the participants think that, as much as the envious character dislikes not being chosen as a representative, he/she could not be happy because his/her peer loses in the competition against others who are not from his/her group. In this case, social identity, that is, the awareness of being part of a group (the same to which the envied belongs) and identification with it, would prevail over personal identity and, therefore, envy and *schadenfreude* would transform into discomfort with the misfortune suffered by a member of the in-group.

The objective of the present study is to explore this possibility, analyzing the influence that making group membership salient has on the emotional and behavioral reaction to an upward social comparison.

### The Present Study

This study aims to investigate what type of comparison context favors the emotion of envy among schoolers. In an interpersonal context, envy arises from the comparison with a similar individual who has the advantage that I want and do not have. If the individual turns out to be part of my group, is envy more intense or less? What happens when my “group self” wakes up? Is the intensity of envy the same when another group has the advantage over mine? Which is stronger, in-group favoritism or envy toward a member of the in-group? The objective of this study is, therefore, to find out how school-age children (from 6 to 11 years of age) perceive and express feelings of envy and *schadenfreude* in a situation of group membership, that is, when their social self is activated, instead of their individual self.

To do so, we created four stories in which the participant had to take on the role of one of the characters in four situations of upward comparison. The first scenario was interpersonal, in which the comparison took place without any mention of group membership of the participant or of any of the characters. In the remaining three stories, group identification was promoted in the participant. In the in-group scenario, the participant compares him/herself with someone in his/her group without any reference to an out-group. In the intergroup story, the group to which the participant belongs competes (and loses) with another group. In the mixed scenario, the participant competes with someone from his/her group to later represent the in-group and compete against a member of an out-group. This last condition is a replica of the one used in the study by Recio and Quintanilla (2015).

In order to address benign envy and separate it from non-envious responses, we have created three response profiles, two envy profiles- malicious and benign—and one without envy. These profiles have been applied to two types of participant's responses: verbal and behavioral. The first response refers to the emotion attributed to his/her character in different points of the stories. The second refers to the allocation of resources under different conditions.

We wish to emphasize that, while studies on the understanding of envy usually ask their participants to attribute the emotion to a third person (a character), in this study we modified this procedure by making the participant assume the role of the envious character and indicate what his/her emotion is at different times during each scenario. This strategy allows the child to become more involved in the group situations, responding as a member of the group rather than as a mere observer (Nesdale et al., 2017).

Our predictions refer to two main factors: the type of social comparison context, and age. Regarding the first variable, we expect that, in a situation of upward social comparison, children's emotions and behaviors, as well as their reactions when the other's advantage disappears, will vary depending on the context in which they find themselves: interpersonal, group, and/or intergroup.

Specifically, we expect that the malicious envy profile, which comprises a feeling of envy accompanied by *schadenfreude* and a wish to damage the envied in the allocations, will be more likely when the envied is a member of the out-group than when he/she is an in-group member, due to in-group favoritism. If the context of comparison is the group itself and, therefore, the envied is a member of the in-group, we do not expect differences in malicious envy with respect to an interpersonal situation, since the in-group comparison highlights the differences between members over their similarities. In the case of the mixed condition (the envied is a member of the in-group in an intergroup situation), an assimilation effect will take place with the rest of the group and in-group favoritism will prevail, thus we will find fewer responses of malicious envy than in the interpersonal and group situations.

Regarding the profiles of benign envy (discomfort with the envied advantage without joy for his/her misfortune and no intention to harm him/her in the allocations) and non-envy (joy for the envied advantage, sorrow for his/her misfortune and a wish to benefit him/her in the allocations), given that in previous studies both types of responses appear mixed (benign envy as such has not been studied in children), rather than posing hypotheses, our objective in the present study is to break them down and explore how each works in different comparison contexts.

With regard to age, the envious responses, both malicious and benign, are very likely to decrease with age, while the inverse relationship will occur with non-envious responses.

## Method

We designed a mixed factorial study to answer these questions regarding the role that group identification plays in the emotion of envy. The within-subject variable consists of four conditions of upward comparison –interpersonal, group, intergroup, and mixed. The inter-subject variable was age. Gender is considered as control variable.

### Participants

The initial sample was 119 children, but one participant was eliminated because of his inconsistent responses in several conditions. Thus the final sample consisted of 118 children (52 girls) between the ages of 6 and 11 (*M* = 8.24, *DT* = 1.60) years [*N* = 37: 6–7 years old (15 girls); *N* = 47: 8–9 years old (23 girls); *N* = 34: 10–11 years old (14 girls)] from three Spanish schools located in Barcelona, Madrid and Salamanca. From this sample, eight children did not identify with the group in some of the conditions and were eliminated from the analysis for those conditions. Specifically, one child was dropped from the Group condition and seven children were eliminated from the Intergroup condition. Moreover, one child was eliminated from the Interpersonal condition due to inconsistent responses.

### Materials

Four stories were designed through which the child was introduced to the different contexts of upward social comparison considered in the study: the three group (in-group, intergroup, and mixed) contexts and the merely interpersonal context. Each story had two versions to match the gender of the main character with that of the participant. Experimenters told the stories with the help of pictures presented on cardboard. Throughout the narratives, participants, as if they were members of a group, answered questions about their emotional state and that of the characters at different moments of the story, and they were asked to justify their answers. For all conditions, the pattern was the same:

- The participant was induced to assume the role of one of the characters in the story.
- He/She was exposed to a disadvantageous situation compared to another individual, emphasizing the previous similarity between the two, in order to induce envy.
- The other suffered a misfortune that made him/her lose his/her advantage, which could generate *schadenfreude* in the participant.

Finally, the participant had to distribute three different-sized bags among three people: an unknown child, the allegedly envied character, and him/herself (or someone from his/her in-group). The bags contained objects that were attractive for the children and coherent with the story told in each case (balloons, candy, stickers, and colored pencils).

In the Supplementary Material we present the four stories, indicating for each of them the different types of questions formulated according to the scenario presented. The four social comparison scenarios are described below:

1. Interpersonal context: the lucky/unlucky child is another similar individual. The participant competes and loses against another child in a context that does not highlight the existence of a group that would frame the relationship between the two.

2. Group context: the lucky/unlucky child is a member of the in-group. In this case, competition and defeat occurred with a classmate with whom the participant had previously had to cooperate, along with the rest of the group, to achieve a common goal. The identification of the participant with the group was activated in an exclusively group context, that is, no information was given about the existence of any other group.

3. Intergroup context: the lucky/unlucky child is an out-group member. In this condition, it was the entire group that was going to compete, and lose, against another group. Therefore, no comparisons were made between members of the in-group.

4. Mixed context: the lucky/unlucky child is a member of the in-group. In this condition, the participant was made to compete and lose to another member of his/her own group to represent the group in a subsequent competition against another group. In this case, two contexts were combined, group and intergroup: the participant, as an individualized member of the group, compared him/herself with another member (contrast effect) and, furthermore, as an interchangeable member (assimilation effect), compared his/her own group with another group.

Participant's identification with the group was induced by informing him/her of his/her belonging to that group. This strategy is usual in studies that employ the minimal group paradigm, where the mere assignment to a group and the information of the existence of an out-group are sufficient for individuals to behave as group members (e.g., Yee and Brown, 1992; Spielman, 2000; Nesdale and Flesser, 2001; Nesdale et al., 2007; Dunham et al., 2011). In the group condition, in the absence of an out-group, identification was induced by highlighting the interdependence between group members and the fact that group success had been achieved thanks to the cooperation among all members (Sherif et al., 1961; Johnson and Johnson, 2005).

### Measures

We took measurements of verbal emotional reaction of (1) the participating child faced with his/her own disadvantage and with the misfortune of the other, and (2) the envied character faced with his/her own advantage and subsequent misfortune, as well as the behavioral reaction of the participant in the final resource allocation task. We also measured participant's identification with the group.

#### Verbal Responses

We recorded the emotional response of the participating child and of the envied character [faced with their own (dis)advantage and subsequent misfortune], and obtained the emotion valence and intensity. Children's responses to the emotional attribution questions (about how they felt and how the envied character felt) were open-ended and expressed different emotions that were simply coded as “Good” for the positive (happy, pleased, proud, etc.), and as “Bad” for the negative ones (sad, unhappy, angry, etc.). To measure the emotional intensity in the different situations posed by each story, we used a scale consisting of three circles of different sizes. The attribution of emotion to the envied character (given his/her initial advantage and later misfortune) served as a control of participants' emotional knowledge; their emotional reaction to their own disadvantage and the subsequent misfortune of the other served to characterize their response pattern as envious or non-envious.

#### Behavioral Responses

In the final resource allocation task, we recorded to whom the child assigned each of the three bags and the justification he/she gave for his/her way of distributing the resources.

#### Response Profiles

Table 1 shows the definition of the three profiles created to classify the verbal (when faced with one's own disadvantage and the misfortune of the other) and the behavioral (in the final resource allocation task) reactions of the participating child. Verbal reactions were composed of two types of responses: a qualitative one, emotional valence (*how do you feel about the advantage/disadvantage of the envied person?*), and a quantitative response about intensity of the emotion (*how good/bad?*). The verbal emotional profile is based on the combination of the emotional valence of responses to these two questions. Thus, if the participant gives a negative emotional response (bad, sad, angry, envious, etc.) to the benefit obtained by another and a positive emotional response (happy, joyful, pleased, delighted, etc.) to the loss of the other's advantage, he/she gives a malicious envious response. Whereas a combination of a negative emotional response (feeling bad for the benefit of another) plus a second negative emotional response (feeling bad for the loss of the benefit) is considered benign envy, and finally, a combination of a positive response to the other's success and a negative response to the loss of the benefit is a non-envious response. The quantitative part of the profile is provided by the intensity score the participant indicates from 1 to 3 for each question (how bad/good). Therefore, the two scores are added up and the range of each profile is from 2 to 6. To simplify the analysis we have converted the intensity into a dichotomous variable: low (≤ 3), and high (≥4). This was carried out for each of the four conditions/stories in the study.

**Table 1:** Definition of the envious and non-envious response profiles for verbal and behavioral responses.

	**Emotion when faced with the other's advantage (verbal)**		**Emotion when faced with the other's misfortune (verbal)**		**Treatment toward the envied during the resource allocation task (behavioral)**		
**Profile**	**Response**	**Intensity**	**Response**	**Intensity**	**Response**	**Allocation for the envied**	**Allocation for the participant**
Malicious envy	BAD	Between 1 and 3	GOOD (*Schadenfreude*)	Between 1 and 3	Harmful	Small bag	Big bag^*^ or Medium bag^**^
Benign envy	BAD	Between 1 and 3	BAD	Between 1 and 3	Nor harmful nor beneficial	Medium bag	Big bag^*^ or small bag^**^
Non-envious	GOOD	Between 1 and 3	BAD	Between 1 and 3	Beneficial	Big bag	Small bag^*^ or Medium bag^**^

* *Allocation score = high.*

** *Allocation score = low*.

For the behavioral measure we also defined three profiles based on the size of the gift allocated to the envied character, and two scores for each of the profiles, high or low, depending on the size of the gift kept by the participant. Thus, a high malicious envy allocation profile meant that the envied received the worst part (the smallest bag) of the allocation, while the participant kept the best (the largest); the low malicious profile meant that the participant kept the medium bag. In the high benign profile, the envied had the medium-sized bag (more generous than high malicious) and the participant kept the largest one; in the low benign profile the participant kept the small one. Finally, a non-envious assignment (generous toward the envied) with high profile was one whereby the envied received the largest bag and the participant kept the smallest bag; in the low profile the participant kept the medium-sized bag (see Table 1).

#### Identification With the Group

As can be observed in the Supplementary Material, for the three conditions that imply a group context, identification control questions have been included (“Do you like being a part of this class/of this school/of the pirates?,” “How much?,” “Would you like to move to another class/another school/be a part of the robots?,” “How much?”). In addition to these questions, we considered the consistency between the emotion that the child says he/she feels and the emotion he/she attributes to his/her group. Moreover, in the case of the Intergroup situation, the fact of favoring the in-group member in the allocation task was taken into account. In the Mixed condition, one more response was considered as an identification indicator: choosing the member of the in-group when asked who he/she wants to win the race (his/her partner or the student from the other school). These indicators allowed us to ensure that the participant responded from the perspective of a group member^1^.

### Procedure

To carry out the study, we contacted the directors of the schools, and obtained informed consent from the parents. Three trained interviewers, one for each center, collected the data. They carried out the interviews individually, within school hours, in a room that each school had reserved for such purpose. After introducing him/herself and obtaining the participant's assent, the interviewer explained the activity. Before starting with the storytelling, brief familiarization training was carried out with the circle scale that was going to be used to measure the intensity of the emotions. Each participant listened to the four stories and answered the corresponding questions related to identification with his/her group, his/her own emotions and those attributed to the other character, and the allocation of the three bags (see Supplementary Material). While the participant carried out the allocation, the interviewer pretended to be distracted by looking away, to avoid making the participant feel pressured; once the allocation was completed, the child would notify the interviewer, who would then ask the child to explain why he/she had allocated the prizes in this way. In total, the time spent with each participant was ~20 min.

The stories were always told in the same order: 1. Interpersonal, 2. Group, 3. Intergroup, and 4. Mixed. The interpersonal situation had to appear before any other to avoid any group or intergroup inference that would make the interpretation of the results difficult. In the same way, the group situation had to precede those involving an out-group. The objective was to prevent the memory of elements from one situation from interfering with the responses to the next situation, as each participant went through all the conditions. The data were collected manually on record sheets designed for the study.

The UNED Bioethics Committee approved the research and its method.

### Data Analyses

To perform the quality control of the data obtained, we carried out the corresponding descriptive analysis, and examined the degree of emotional knowledge shown by the participants, as well as their identification with the reference group. In this way, we could detect possible participants with an anomalous response pattern, that is, those who exhibit problems in correctly attributing the emotion to the envied character, or difficulties in identifying with their group. Likewise, the possible effects of the interviewer and the participant's gender were examined, performing the corresponding χ^2^-tests on the measures with verbal and behavioral responses, which did not reveal significant differences (all *p* > 0.05).

Considering that the variables are dichotomous in terms of intensity (high and low) for each verbal and behavioral response profile, we have conducted non-parametric analyses (chi-square test) that allow us to test the hypotheses about the effects of age and experimental conditions.

The justifications referring to the allocations were classified into categories using an inductive analysis. Two researchers performed the coding independently, and a high reliability was obtained (κ = 0.96, *p* < 0.001).

## Results

All the participants showed emotional knowledge appropriate for the situations of the characters, and attributed emotions to the envied character according to the circumstances of success or failure.

A first descriptive analysis of the matches between the three verbal and the three behavioral response profiles found < 50% matches between the two forms of responses across the four experimental conditions. This can be seen in Table 2, where the values on the diagonal represent the matches for each condition. As an extreme example, in the Mixed condition, no child gave a maliciously envious verbal response, while 12 participants allocated in a maliciously envious way. It was also striking that there were more non-envious shares than verbal responses in this same profile.

**Table 2:** Frequencies of participants in verbal and behavioral profiles.

	**Verbal profiles**							
	**Interpersonal**				**Group**			
**Allocation profiles**	**Malicious**	**Benign**	**No envy**	**Total**	**Malicious**	**Benign**	**No envy**	**Total**
Malicious	5	8	3	16	7	11	1	19
Benign	8	22	6	36	5	20	14	39
No envy	4	36	25	65	4	32	23	59
Total	17	66	34	117	16	63	38	117
	**Intergroup**				**Mixed**			
Malicious	6	16	1	23	0	9	3	12
Benign	4	22	6	32	0	34	10	44
No envy	14	33	9	56	0	38	24	62
Total	24	71	16	111	0	81	37	118

Therefore, we considered working with the verbal and the behavioral response profiles separately, taking as the unit of analysis the frequencies of responses obtained in each condition.

### Verbal Profiles by Experimental Conditions and Age Groups

#### Effect of Experimental Conditions

The verbal profile of malicious envy has the lowest frequency of response of the three profiles. The total scores of emotional intensity are similar for high and low intensities. Only three children had consistent malicious envy responses in all conditions except for the Mixed one, and 15 children had this profile in two conditions; the rest (39) had it in only one condition. This profile is clearly influenced by the experimental conditions. We can see in Table 3 that the Mixed condition has no malicious envy verbal responses, in contrast to the other three conditions. The chi-square test is less reliable when it contains cells with scores equal to 0 or <5. Therefore, to find out whether the conditions affected the intensity of the emotion expressed, we compared only three conditions: Interpersonal, Group, and Intergroup. The results revealed no significant association between intensity and conditions. Our results seem to indicate, in line with our predictions, that upward social comparison elicits more malicious envy when the upper hand is held by an out-group in a context of intergroup conflict. However, we cannot assert this statistically because the Mixed condition is left out of the analysis.

**Table 3:** Frequency distribution of the verbal profiles in each experimental condition.

**Profiles**	**Interpersonal**	**Group**	**Intergroup**	**Mixed**	**Total**	**Chi-square**
**Malicious**						χ^2^(2) = 0.76, *p = 0.68*
Low	7	8	13	0	28	
High	10	7	11	0	28	
Total	17	15	24	0	56	
**Benign**						χ^2^(3) = 26.78, *p* =0.001, 1- β = 0.96, w = 0.33
Low	47	34	25	26	132	
High	19	29	43	55	146	
Total	66	63	68	81	278	
**No envy**						χ^2^(3) = 9.95, *p* =0.02, 1- β = 0.95, w = 0.30
Low	15	12	9	7	43	
High	18	25	6	30	80	
Total	33	37	15	37	122	

*The frequencies correspond to the total responses obtained from the sample*.

The benign envy profile has a higher response frequency than the other two profiles. The frequency of emotional intensities is differentially distributed among conditions. High intensity responses of benign envy were most frequent in the Intergroup and the Mixed conditions, while the highest frequencies of low intensity are found in the Interpersonal and the Group conditions (Table 3). Only 23 participants were consistent in their benign responses to all conditions. There were 109 participants that gave responses according to this profile at least in one of the conditions, but not in all of them. The result of the chi-square test indicated that the conditions were associated with the differences among the intensities of benign envy with a medium effect size. This result reinforces the idea that the presence of an out-group protects against malicious envy toward a member of the in-group. Benign envy, at least in terms of its verbal expression, is most frequent, in its highest intensity, in the Mixed condition, while in the case of malicious envy the frequencies for this condition were 0.

The no-envy profile also contains different frequencies in each experimental condition. The intergroup condition had the lowest total frequency of no envy. The association among conditions and intensity was significant. As we can see in Table 3, in the Mixed and the Group conditions, where the envied is from my group, the frequencies of non-envious high response profile increase, to a greater extent in the Mixed condition, probably due to the presence of an external group.

#### Effect of Age

Table 4 represents the frequencies of the profiles with high and low emotional intensity for the three age groups. Results of the chi-square test indicate that emotional intensity is not associated with age groups in the two envious profiles but it is in the no-envy profile. As can be seen, non-envious responses increase with children's age, particularly between six-seven and eight-nine years.

**Table 4:** Frequency distribution of verbal profiles of responses by age group.

**Profiles**	**Age groups**			**Total**	***Chi-square***
	**6 years**	**8 years**	**10 years**		
**Malicious**					χ^2^(2) = 0.30, *p* = 0.86
Low	11	11	7	29	
High	9	11	7	27	
Total	20	22	14	56	
**Benign**					χ^2^(2) = 4.52, *p* = 0.10
Low	36	55	41	132	
High	55	59	32	146	
Total	91	114	73	278	
**No envy**					χ^2^(2) = 10.73, *p* = 0.004, 1-β = 0.99, w = 0.31
Low	11	17	31	59	
High	18	30	15	63	
Total	29	47	46	122	

Summarizing our results for verbal responses, we found that experimental conditions did have an effect on the type and intensity of emotion expressed by participants. Confirming our predictions, malicious envy disappears in the Mixed condition. However, for the benign envy profile this condition shows higher frequencies of high emotional intensity than the other three. As for the Group condition, also supporting our predictions, frequencies of malicious envy are very similar to those of the Interpersonal condition. Moreover, as expected, the Intergroup condition shows higher total frequencies in the malicious envy profile than the other three conditions (albeit only as a tendency), and a very low total frequency in the profile of no-envy. Finally, contrary to our expectations, the emotional intensity of the verbal envious profiles was not associated with age. On the other hand, we did find an increase of non-envious responses with age.

### Treat Allocations

The analysis of the treat allocations first compared the differences of the type of allocation among experimental conditions. We will subsequently report our results regarding differences among age groups.

The allocations of the three high and low profiles in the four conditions are shown in Table 5. It is interesting to note that while the Mixed condition in the verbal profile of malicious envy obtained zero frequencies, in the behavioral profile of this same condition there were 12 maliciously envious responses. In general terms, malicious allocations are more frequent in the high profile than in the low profile in the first three conditions (Interpersonal, Group and Intergroup). These differences, as shown in Table 5 are significant (small effect size).

**Table 5:** Distribution frequencies of the total allocations according to the experimental conditions and Chi-square results.

**Profiles**	**Interpersonal**	**Group**	**Intergroup**	**Mixed**	**Total**	***Chi-square***
**Malicious**						χ^2^(3) = 8.6, *p* = 0.03 1−β = 0.58, w = 0.27
Low	5	8	1	6	20	
High	11	11	22	6	50	
Total	16	19	23	12	70	
**Benign**						χ^2^(3) = 17.09, *p* <0.001 1−β = 0.99, w = 0.37
Low	13	21	9	31	74	
High	23	18	24	13	78	
Total	36	39	33	44	152	
**No envy**						χ^2^(3) = 19.15, *p* <0.001 1−β = 0.95, w = 0.30
Low	33	39	42	24	138	
High	33	20	14	38	105	
Total	66	59	56	62	243	

Allocations with benign envy profiles are distributed differently, with the high profile (the envier keeps the biggest gift and the envied gets the middle one) being more frequent in the Interpersonal and Intergroup conditions, and the low profile (the participant gets the smaller one) being more frequent in the Mixed and the Group conditions. These differences are significant, with a medium effect size.

The non-envious shares are more frequent in the low profile than in the high profile in the Group and the Intergroup conditions, while the high profile of non-enviousness is most frequent in the Mixed condition. These differences in the distributions are statistically significant.

Regarding the age factor, as we can see in Table 6, high malicious envy profile predominates in 6- and 8-year-old participants, and decreases in the older ones. These differences are significant. A similar pattern can be found in the benign envy profile. High intensity (when the participant keeps the largest bag) is most frequent in the 6-year-old group, 8-year-olds show similar low and high profile frequencies, and the older age group has higher frequencies in the low than in the high profile. These differences are also significant. On the contrary, in the non-envious profile, low intensity (when participant keeps the medium-sized bag instead of the small one) is more frequent than high intensity for the three age groups. In this case, differences are not significant. All these chi-square results about differences have small effect sizes (see Table 6).

**Table 6:** High and low allocation profiles by age groups and results of Chi-square test.

**Profiles**	**Age groups**			**Total**	***Chi-square***
	**6 years**	**8 years**	**10 years**		
**Malicious**					χ^2^(2) =10.45, *p* = 0.005 1−β = 0.95, w = 0.30
Low	7	5	8	20	
High	30	16	4	50	
Total	37	21	12	70	
**Benign**					χ^2^(2) = 14.68, *p* <0.001 1−β = 0.99, w = 0.38
Low	20	29	25	74	
High	41	28	9	78	
Total	61	57	34	152	
**No envy**					χ^2^(2) = 0.19, *p* = 0.90
Low	26	63	49	138	
High	21	45	39	105	
Total	47	108	88	243	

In general, and in line with our predictions, a high profile of malicious envy allocation decreases with increasing age, and the same is true for benign envy.

To summarize, our results with the behavioral measure show a different pattern than those with the verbal measure. While maliciously envious responses are still less frequent than those in other profiles, they seem to be more frequent and of higher intensity when children act than when they speak. The Mixed condition is, again, an extreme example, but the same is true for the other conditions. These differences notwithstanding, our predictions are fulfilled in that malicious envy is most frequent and intense in the Intergroup condition and least frequent and intense in the Mixed condition. The reverse is true for non-envious allocations. As for benign envy profile, the highest total frequency corresponds to the Mixed condition, as with the verbal measure, although in this case it is predominantly low in intensity. Regarding the relation between age and allocation profiles, our results support our expectations, in that envious responses (both malicious and benign) decrease as age increases.

### Justification for the Allocations

The behavioral measure is a novel aspect of this study within the area of research on envy in children. We have considered it important to explore the arguments that children use to justify their decisions, because they can provide us with clues regarding the reasoning behind the allocation of resources in a context in which the child is at a disadvantage. Therefore, after the allocation task, participants were asked to explain why they had allocated the bags in that way. We coded and grouped these responses into various categories, which are described in Table 7.

**Table 7:** Response categories for the justification of the allocations (behavioral measure).

**Category**	**Reasoning elements**	**Examples**
Prosocial	Positive orientation toward others, focus on their situation, and allocation benefits them.	“He has hurt himself”; “She is my friend”; “We must be generous.”
Egocentric	Positive orientation toward oneself or negative toward others, and the allocation is selfish.	“They have chosen him instead of me”; “I don't like her”; “I want the big bag.”
Deservingness/Justice	Evaluation (positive or negative) of the person (or group): ability, achievement, good/bad behavior; balance of benefits.	“She has done well”; “She has won”; “He has cheated”; “He has already had an award.”
In-group favoritism^*^	Positive orientation toward the other because he/she is part of the in-group, and the allocation benefits him/her for that reason.	“He belongs to my group”; “I like the pirates (in-group) better.”
Mixed	Reasoning that can be classified into more than one category, none of which are clearly dominant.	“She has been brave, and she belongs to my group”; “He has made our school lose and I don't like him.”
Other	The reasoning does not follow a classifiable criterion.	“She doesn't like balloons”; “He told me he doesn't want candy.”

* *This category is only applicable to the Intergroup and Mixed conditions*.

In order to find out whether the children's arguments varied according to the context in which the relationship between envious and envied is framed, we carried out a qualitative analysis of the justifications. Table 8 contains the frequency distribution of the explanations for the allocation in each condition. The row of subtotals shows the frequencies of the distributions by profile and condition.

**Table 8:** Frequencies of the justification categories in the three types of allocation for the four experimental conditions.

	**Interpersonal**			**Group**		
	**Malicious**	**Benign**	**Non-envious**	**Malicious**	**Benign**	**Non-envious**
Prosocial	0	10	21	0	17	21
Egocentric	9	7	0	7	8	0
Deservingness	1	4	23	2	2	15
Mixed	4	9	19	6	10	20
Other	0	3	1	1	1	1
Subtotal	14	33	64	16	38	57
	**Intergroup**			**Mixed**		
	**Malicious**	**Benign**	**Non-envious**	**Malicious**	**Benign**	**Non-envious**
Prosocial	0	5	4	0	0	5
Egocentric	–	–	–	3	3	0
Deservingness	3	7	19	1	25	9
Mixed	8	14	30	6	13	43
In-group Fav.	9	4	1	0	0	2
Other	2	3	2	1	0	0
Subtotal	22	33	56	11	41	59

As can be seen in Table 8 (subtotal rows), all conditions show a predominance of non-envious allocations over envious ones. Malicious allocation is the least frequent. The differences in the frequency distribution of justifications among the four conditions are not significant (χ^2^(6) = 0.42, *p* > 0.05). It is noteworthy that there are no more non-envious distributions in the conditions in which these would benefit a member of the in-group (that is, the Group and the Mixed) than in the Intergroup condition, where the beneficiary is from an outgroup.

In the justifications related to the allocation of malicious envy, which is detrimental to the envied, the arguments most mentioned in the Interpersonal and Group conditions were of an egocentric type, and in the Mixed condition, this reasoning appeared together with that of deservingness (of the envious, in this case) in mixed justifications (see Table S1 in Supplementary Materials). This reflects a predominance of the personal self over the social self when deciding how to allocate resources. In the Intergroup condition, which showed the highest frequency of maliciously envious allocation, the egocentric criterion gave way to arguments based on in-group favoritism (9) (logical, taking into account that the participant does not enter into the distribution in this case), and mixed reasoning (8) also included this type of justification along with others based on merit and justice, which justify the punishment of the envied outgroup as a compensation to the in-group for not having obtained the prize.

In relation to the benign envy distributions, where the envied is not punished with the worst allocation, but neither is he/she favored, prosocial arguments stand out, either alone or combined with egocentric or deservingness criteria in the Interpersonal and Group conditions. In the Mixed condition, the punishment of the envied member of the in-group was softened according to criteria of merit, as well as a combination of egocentric arguments and in-group favoritism. In the Intergroup condition, egocentrism made no sense again, and in-group favoritism and merit reached a higher frequency, either alone or combined, as it is not the participant who is involved in the allocation but rather a member of his/her group. Here, deservingness is again the criterion that softens the envious response.

In the non-envious allocations of the four conditions, merit and prosocial arguments predominated, either isolated or in combination, which is logical considering that this type of allocation benefits the envied, that is, the one who had won in the beginning and then suffered a misfortune. In the Intergroup condition, the non-envious allocation is mainly justified by deservingness. Children seemed to value this criterion highly, regardless of whether the winner was from their own group or from an out-group. It is also striking that, in the Mixed condition, there were hardly any justifications within the prosocial and in-group favoritism categories (although this type of arguments were mentioned in the mixed justifications), despite the fact that the beneficiary of the allocation in this case would be an in-group member.

## Discussion

This study aimed to analyze the role of group identity in the appearance of envy. Three response profiles were defined whose scores indicated to what extent malicious envy, benign envy, and non-envious responses were produced in four experimental conditions. In three of these conditions, Group, Intergroup, and Mixed (group plus intergroup), the participant was induced to identify with a group, while in the Interpersonal condition emphasizing a group situation was avoided. This allowed us not only to compare our results with those obtained in previous studies on envy in children, always carried out in interpersonal contexts (Steinbeis and Singer, 2013; Recio and Quintanilla, 2015; Jensen de López and Quintanilla, 2019), or considered as such, but also to find out how group identification influences the emotion of envy and its expression.

From a methodological point of view, there are two novel aspects to emphasize in the design of this study: (1) the active induction of group membership in the participant, and (2) the use of three types of measures: attribution of emotion to the characters (emotional valence), estimation of emotional intensity, and behavioral measurement (allocation of resources).

In this respect it is worth stressing the differences between verbal expression of the emotion and resource allocation behavior. Most children generally follow the explicit norm of not expressing malicious envy, and some are inconsistent in their behavior. Another inconsistency occurs when older children express benign envy but allocate in a non-envious way. The first inconsistency may be due to verbal expression being better controlled than behavior. It is easier to say that one is not envious than sharing without envy. Children at this stage in development already know display rules. The second inconsistency has likely a different meaning: Benign envy is expressed openly; this implies acknowledging one's own shortcomings, but sharing without envy may be an indication that the child masters the feeling of envy.

### Comparison Context and Envy

The experimental conditions led to differences in response profiles, both in the verbal and in the behavioral measures. In line with our predictions, the Intergroup condition, where the envied is a competing out-group, shows the highest scores for malicious envy in both measures, as well as the lowest scores for no-envy, and the opposite is true for the Mixed condition, where the envied is an in-group member in an intergroup competition. It seems that the expression of this harsher or more socially penalized type of envy is more justifiable for children when the envied is from an out-group. Moreover, benignly envious responses are most frequent and most emotionally intense in the Mixed condition. Apart from these similarities between the verbal and behavioral measures, we have found several differences which support our decision to analyze the two types of response separately. In general, although malicious envy is the least frequent profile, both in verbal and in behavioral measures, allocations show higher frequencies and higher intensity than verbal responses in this profile, which can be attributable to the fact that behavior is more difficult to hide or regulate than verbal reactions are. The reverse is true for the benign envy profile, the one with highest frequencies in the verbal measure but not so in the behavioral one. It seems as if children are conscious that reporting a benign version of envy, one where you do not rejoice at the others' misfortune, is acceptable enough.

The Mixed condition is particularly interesting because it shows quite clearly how children manage the expression of their envy. Verbally, they transform a malicious version of the emotion into a benign one. Apparently, the conflict with an out-group does not completely eliminate the discomfort felt with the advantage of an in-group partner, although it may counteract more the malicious part of envy, that envy that entails harming the other, as suggested by Duffy et al. (2012). However, this seems to apply more to verbal responses than to behavior. While the malicious verbal profile disappears in this condition, this does not happen with the behavioral measure, where nine children who reported to feel bad when the envied suffered the misfortune, and three children who said they felt happy for the envied person's success allocated the gifts in a maliciously envious way.

If we compare the treatment that participants gave to the envied in-group member in the presence of a competing out-group (Mixed condition) and in its absence (Group condition), the distribution of frequencies show that children show more and more intense maliciously envious responses in this latter condition. These results are congruent with the postulates of the self-categorization theory (Turner et al., 1987): the group situation accentuated the social comparison between two members of the group within a strictly group framework, which enhanced the contrast effect between members and personal identity over social identity. On the other hand, in the mixed scenario, the comparison between members of the in-group occurred in a context of competition with an out-group, which highlighted the social self and promoted an assimilation effect among members that counteracted the feeling of malicious envy. Nevertheless, according to our results, this assimilation effect is not strong enough to completely eliminate upset due to an in-group member's advantage, at least at these age levels. At the most, it changes into a milder version of envy.

One of the novel elements that we must highlight from the present study is that it provides a measure of benign envy, defined according to previous studies that delimit this concept (Van de Ven et al., 2015) from the perspective of the development of emotional understanding. Without this measure, every response not classifiable as malicious envy would have been ruled out as non-envious. We thus contribute toward separating this type of emotion from that which was considered in previous studies in an undifferentiated sense as the absence of malicious envy. This is a step forward that may add to a more parsimonious study on the development of understanding of envy in children.

### Age Differences

The results revealed two different patterns of responses in verbal and behavioral measures. Age did not influence scores on the two envious verbal profiles, probably because by this stage in development children have already acquired emotional display rules that prevent them to show envy explicitly and promote other more socially acceptable reactions. However, younger children showed significantly fewer non-envious verbal responses than the other two age groups. Regarding behavioral responses, there were more maliciously and benignly envious allocations in six-seven and in eight-nine age groups than in the 10–11 age group. Non-envious allocations were non-significant in relation to age.

This interesting result seems to indicate that at least from the age of 6 years children may hide their envious verbal responses rather than their actions involving envious sharing. Studies with children aged 3–8 years, comparing knowledge of sharing rules and behavior, show a gap between what young children know about the rules and how well they follow them when sharing with others. Their distributions are interested even though they know the rules; this gap fades with increasing age (Smith et al., 2013). In our study, although with a different goal, this gap between the norm of not showing a maliciously envious emotion and the action of self-interested sharing is also present. However, as we have seen, the envious distributions (malicious and benign), in general terms, have lower frequencies than the non-envious allocations.

These results coincide with those found in previous studies in which, from 6 years onwards, envy progressively disappears. But we must stress that this coincidence apply specifically to malicious envy, because benign envy, the expression of which is socially accepted, appears at the ages of six and eight, and less frequently at the age of 10. The question is: does this have to do with social desirability?

The so-called social desirability is understood as a “threat” in most self-report studies, whose presence in the responses compromises the validity of the construct. However, within a developmental context, we could interpret social desirability as a social and cognitive achievement, reflecting the child's ability to understand display rules. As some studies reveal, the display rules that are part of socio-emotional skills are manifested around the age of 8 (Broomfield et al., 2002; Cheung et al., 2015). In the present study, appearing non-envious or benignly envious can be a way of displaying children's knowledge of acquired display rules and other social norms.

From a child development perspective and that of the strictest demands of social life, it is important to consider these socially desirable responses more like an achievement than a “methodological threat” in this social situation, as it implies that the child, from the age of six, understands that providing appropriate emotional responses fosters a good self-image and keeps social relationships in harmony in situations where it is socially normative to inhibit (or not express) envy and *schadenfreude*. In this sense, and in general terms, our results indicate that children between the ages of 8 and 10 years show fewer socially undesirable responses to the misfortune of others, such as malicious envy. This type of response is congruent with studies that find that children prefer, in social comparison scenarios, modest attitudes (of self-contempt) to envious ones (contempt toward the individual with the advantage) from the age of 8 years, but at 6 years of age, they can already recognize that envious responses are not socially acceptable (Quintanilla and Giménez-Dasí, 2017; Quintanilla et al., 2018). They also are in line with studies that find a relationship between the progressive decrease in feelings of envy and *schadenfreude* and the increase in egalitarian decisions in the allocation of resources (e.g., Steinbeis and Singer, 2013), which some authors relate to theory of mind abilities and empathic perspective taking (e.g., Cowell et al., 2016). Nevertheless, although the decline in envious responses with age is a pattern confirmed in numerous studies, it has also been empirically proven that both envy and *schadenfreude* are emotions present in adults (e.g., Takahashi et al., 2009; Dvash et al., 2010). As Steinbeis and Singer (2013) suggest, it is likely that what evolves with age is not so much the actual experience of these emotions but rather the ability to regulate them.

### Reasoning Behind the Allocation of Resources

The results of the qualitative analysis show that the arguments that children use when justifying their allocation decisions are often mixed, that is, they use more than one criterion to decide. The high frequency of deservingness, both alone and in combination with other criteria, indicates that, despite showing envy toward the other, they recognize his/her superiority and express that recognition by benefiting (or, at least, not penalizing) him/her in the allocation (e.g., “*because they won”; “because they deserved the award”; “because my drawing was not chosen”*). The importance that children confer to merit and justice is related to the aversion toward inequality (Fehr et al., 2008; Moore, 2009; Blake and McAuliffe, 2011). In this sense, it is possible that knowing that the envied has lost his/her advantage due to the misfortune makes the children perceive that, in a way, the inequality has been resolved, and they focus more on the merits of the other. In the Intergroup condition, the consideration of merit seems to be the one that mostly contributes toward increasing the score of the non-envious allocation, more so than prosocial arguments.

A closer inspection of Table 8 suggests that children use their arguments strategically, as appropriate to justify their decisions. In some cases, the merit is attributed to the envied (“*S/he has made a lot of effort, and will feel very bad*”), and in others it is attributed to oneself (“*because I have done well*”; “*I draw well, too*”) or the in-group (“*Because we did better than the robots*”). In the same way, it is striking that in-group favoritism (“*Because s/he is from my group*”), which should predominate in both the Intergroup and Mixed conditions, in the latter condition only appears combined with egocentric arguments to justify the allocations of benign envy. What these results seem to indicate is that upward social comparison with a member of the in-group activates the personal self and provokes a feeling of envy, which would be mitigated (taking its benign form) by the in-group favoritism provoked by competition with an outgroup that activates the social self. In short, it seems that the relationship between envy and in-group favoritism is complex and bidirectional. On the other hand, it is necessary to consider the possibility that sometimes the participants are “inventing” the justifications *a posteriori*, and that these do not always really reflect the cognitions used in the allocation decisions. Children may know what the socially prescribed norms are in a particular situation and may not follow those norms when they harm them (Smith et al., 2013).

### Practical Implications

Our results have also an applied significance. Studies with adults have repeatedly shown that making salient a group identity fosters trust and cooperation where individuals tend to compete, for example, in social dilemmas (e.g., Brewer and Kramer, 1986; Dawes et al., 1988; Caporael et al., 1989). Furthermore, group identification seems to be negatively related to feelings of envy toward in-group members (e.g., Brewer and Weber, 1994; McFarland and Buehler, 1995; Stapel and Koomen, 2001; Gardner et al., 2002; Chen and Li, 2009). In our study, where envy can be considered as a consequence of a competition between the envier and the envied, the strong version of envy is particularly low when the envier considers the envied as an in-group member and there is a competing out-group. The presence of an outgroup probably had the consequence of accentuating the assimilation effect with other group members (Turner et al., 1987). Thus, in order to help children manage maliciously envious feelings and behaviors, a useful strategy could be to stress common identity, common bonds and goals between children. In this way, an upward social comparison with a group member would, at the most, elicit benign envy, which is undeniably positive in many ways (Duffy et al., 2012; Van de Ven, 2017; Van de Ven and Zeelenberg, 2020). Intervention programs aimed at improving socioemotional competencies in children could be enriched by incorporating strategic activities to manage emotions and improve the social climate of the classroom.

### Limitations

Although our results indicate that children have successfully identified with their role in the stories, have answered as members of a group and have reacted with or without envy depending on the experimental conditions, the present study has some limitations that need to be highlighted.

An important limitation lies in the fact that the scenarios herein considered differ in aspects that could be affecting the results (e.g., who chooses the winner, between whom the allocation is carried out). It is probably not the same that the in-group chooses the winner among its members as if the decision comes from an external jury. In the first case, not being chosen can be interpreted as a rejection by the group, while in the second that implication would not exist. Also, the fact that the participant him/herself is or is not a potential beneficiary of the allocation of prizes can influence the result.

Furthermore, participants are forced to allocate treats on an unequal basis, which might be limiting their choices and make it difficult to interpret their decisions. Normally, inequality aversion is assessed by making children share out resources which are equal-sized portions to see whether they distribute them according to the criteria of fairness or equality (Fehr et al., 2008; Shaw and Olson, 2011). In our study, one might think that non-envious allocations are the product of inequality aversion, but we cannot be sure because the distribution was inevitably unequal. It would be interesting to use equal-sized portions, and in a larger amount than individuals to receive them. Thus, the dilemma for the participant would be to decide whether someone or no one gets the extra portion. This would provide evidence of inequality aversion. Although none of these limitations affects the hypotheses raised, in future studies it would be convenient to develop more homogeneous scenarios that allow the emotions involved to be discriminated more clearly.

A question that would also be interesting to explore is the extent to which children attribute malicious envy to the rest of the group while avoiding doing it to their own character, as a strategy to maintain a socially acceptable image. Our data points in that direction, but a more thorough analysis is necessary to be able to extract conclusive results.

## Conclusions

Group context, and especially the presence of a competing outgroup, influences the appearance of envious feelings (benign and malicious envy) in situations of upward social comparison. When the child identifies him/herself with the group and the one who has the advantage is a member of the in-group, envy decreases in contexts of intergroup competition, and the opposite occurs when the advantage corresponds to an outgroup member. With age, envious responses decrease. This evolution might indicate that children are acquiring display rules, and one of those rules is to show themselves appropriately to others, probably not to harm them, but also to safeguard their own public image. In this sense, benign envy, being a form of envy that is more socially acceptable because it lacks the objectionable component of *schadenfreude*, might be performing a balancing function between public image and individual interest. Nevertheless, this balance seems more apparent in children's verbal expressions than in their behavior.

## Data Availability Statement

The raw data supporting the conclusions of this article will be made available by the authors, without undue reservation.

## Ethics Statement

The studies involving human participants were reviewed and approved by Comité de Bioética de la Universidad Nacional de Educación a Distancia, Vicerrectorado de Investigación, Transferencia del Conocimiento y Divulgación Científica, UNED, Spain. Written informed consent to participate in this study was provided by the participants' legal guardian/next of kin.

## Author Contributions

EG: conceptualization, methodology, writing-original draft, and writing, review and editing. MN: methodology and formal analysis. LQ: conceptualization, methodology, formal analysis, and writing-original draft. All authors contributed to the article and approved the submitted version.

## Conflict of Interest

The authors declare that the research was conducted in the absence of any commercial or financial relationships that could be construed as a potential conflict of interest.

## Footnotes

^1^An indirect measure of identification was obtained through the children's explanations in the open-ended questions. For example, when they used the term “we” when talking about the group, when they referred to the group's failure as their own failure, or when they referred to the ties that linked them with the group. However, although these types of responses were frequent, none of them were taken into account in order to include or exclude participants from the analyses.
