# Supplementary material for: Influence of Group Identification on Malicious and Benign Envy: A Cross-Sectional Developmental Study
Source: Front Psychol. 2021 Jun 30;12:663735. doi: 10.3389/fpsyg.2021.663735 (PMC8277992; doi:10.3389/fpsyg.2021.663735)
Supplement: Supplementary file 1 [file Data_Sheet_1.pdf]

Stories corresponding to the four experimental conditions

General instructions

Experimenter: "*Look, I'm going to tell you four imaginary stories. As I tell you about them, I want you to imagine that you are the main character. Then, I will ask you some questions to know how you have felt and your opinions. I ask you to be sincere in your answers. There are no good or bad answers, they are all valid, as long as they are what you really think. Are you willing to help me?* (If he/she does not accept, he/she is thanked and another child is asked*) Great, thank you for your collaboration! Let's get started then*".

Interpersonal Story

First scene
"The teacher has told you that they will give a great prize in the next drawing contest. Also, the best drawings will be displayed in an exhibition for everybody to see. The class will choose the drawing that is to take part in the contest.
Everyone in the class knows that John/Mary and you draw very well. You both want your drawing to be chosen".
Second scene
"The class has chosen John/Mary's drawing. And it is so good that it wins the prize in the contest"
Emotional attribution questions
1. "*How does John/Mary feel when his/her drawing is chosen and wins the prize*?"
2. "*And you, how do you feel when they choose John/Mary's drawing instead of yours and you don't get the prize?*"
3. "*How good/bad?"* (scale)
4. "*Why?"*

Third scene
"Everything is ready to present the drawings at the exhibition. All the pictures are on the board, but John/Mary's drawing has gotten wet. Now they can't put it on display."
Emotional attribution questions
5. "How does John/Mary feel when he/she sees that his/her drawing has gotten wet and he/she can no longer participate in the exhibition?"
6. "How good/bad?"
7. "And you, how do you feel when you see that John/Mary's drawing has gotten wet?"
8. "How good/bad?"

Allocation task
"Do you remember what happened in this story? The class did not choose your drawing and you could not compete. They chose John/Mary's drawing, which won the award. Then, the drawing got wet and they couldn't put it on display.
*Look, after the exhibition, these colored pencils are handed out. You are asked to share them out. You only have these three boxes of colored pencils left; do you see them? Some boxes are larger than others. And only these children haven't received theirs yet (*the participant is shown a picture with three drawn faces*): John/Mary, a boy/girl you don't know and you. You decide how you will share the pencils out, and I'm not going to tell them that it was you who decided."*
The experimenter says, before giving the participant the pencil boxes: "*While you*

distribute these, I am going to look in my folder for some papers that I need. When you're done, let me know, okay?" Then, the experimenter gives the participant the pencil boxes. He turns around and searches the folder without looking at how the participant allocates the pencils. When the participant says that he/she is done, the experimenter turns around and asks:
9. "Which one did you give John/Mary?" "Why?"
11. "The boy/girl you don't know, which one did he/she get?" "Why?"
13. "And you, what box did you keep?" "Why?"

Group story

First scene
"Your class really likes music. The parents' festival at the end of the year is approaching, and you have painted a beautiful mural together in class.
The teachers liked your mural so much that they decided to use it as a decoration at the festival. In addition, the school director will give a diploma to the class for your good work."
Identification control questions
1. "Do you like being a part of this class?"
2. "How much do you like it?"
3. "Would you like to move to another class?"
4. "How much would you like it?"
Second scene
"You, like everyone in your class, really want to sing at the festival. But only one will be chosen and he/she will be who will collect the class trophy. Peter/Paula and you are the ones who sing the best at the audition."
"The class chooses Peter/Paula."
Emotional attribution questions
5. "How does Peter/Paula feel when he/she gets chosen?"
6. "How good/bad?"
7. "And you, how do you feel when you see that they have chosen Peter/Paula and not you?"
8. "How good/bad?"
9. "Why?"
Third scene
"On the way to the presentation, a car splashes Peter/Paula with mud. He/She returns home to change his/her clothes but lives so far away that he/she does not arrive in time to sing at the festival. No one else knows the song well so there is no music at the presentation. In addition, the teacher collects your class' trophy."
Emotional attribution questions
10. "How does Peter/Paula feel when he/she sees he/she can't get to the presentation?"
11. "How good/bad?"
12. "And you, how do you feel when you see that Peter/Paula can't get to the presentation?"
13. "How good/bad?"
14. "Why?"
15. "And the rest of your classmates, how do they feel when they see Peter/Paula won't make it to the presentation?"
16. "How good/bad?"
17. "Why?"

Allocation task

"Do you remember what happened in this story? They were going to give your class a diploma for the good work you did together with the mural. The class didn't choose you to sing at the performance, so you couldn't sing. They chose Peter / Paula. He/She didn't make it to the performance on time, so he/she couldn't sing or pick up the class diploma.

These balloons are distributed at the end of the festival. You're the one who shares the balloons out and you're almost done. You still have three bags of balloons to share out among the three of you (a picture is shown with the three faces): Peter/Paula, a new boy/a new girl who has just arrived at school, and yourself. You must decide how to distribute them, and I'm not going to tell them that it was you who decided."

"While you distribute them, I am going to send a message on my phone. When you finish, let me know." (The experimenter gives him/her the three bags of balloons and gets up to send the message, with his/her back to the child. When the child calls him/her, the experimenter asks.)

18. "You gave Peter/Paula the ...". "Why?"

19. "You gave the new boy/girl the....". "/Why?"

20. "And you kept the ...". "Why?"

Intergroup story

First scene

"At the carnival party, all the children are preparing their costumes. The group that makes the best costume will win a grand prize. You have the idea that your group can dress up as pirates, and everybody says "Great!!!" The other group will disguise themselves as robots. Pirates and robots want to win the prize."

Identification control questions

1. "Do you like being in the Pirates' group?"

2. "How much?"

3. "Would you like to change over to the robots' group?"

4. "How much?"

5. "Why?"

Second scene

"Everyone really likes robots, because they have lights on their costume. They get lots of applauses as they walk down the street. The jury decides that the robots win the prize."

Emotional attribution questions

6. "When the jury announces their decision, how do the robots feel?"

7. "How good /bad?"

8. "Why?"

9. "And you, how do you feel when you see they give the prize to the robots?"

10. "How good/bad?"

11. "Why?"

12. "And how do the pirates feel?"

13. "How good/bad?"

14. "Why?"

Third scene

"In the parade, the pirates march at the beginning, the robots march behind. Just as your team arrives at the theater where the awards will be given, it starts to rain heavily. Those behind you in the parade get completely wet, and the robot costumes are

spoiled."

Emotional attribution questions

15. "How do the robots feel when their costumes are ruined?"
16. "How good/bad?"
17. "And you, how do you feel when you find out the robots' costumes are ruined?"
18. "How good/bad?"
19. "Why?"
20. "How do the pirates feel when they see that the robots' costumes are ruined?"
21. "How good/bad?"

Allocation task

"Do you remember what happened in this story? The pirates were not chosen in the costume contest. The robots were chosen and won the prize. In the end, the robots' costume was ruined because it started to rain, and they got wet.
The next day…
The carnival is over and now all the sweets that were collected are going to be shared out. It is your job to distribute these three boxes of sweets to the three children who haven't received theirs yet (a picture with three faces is shown: one of a pirate, another of a robot and another of a boy/girl who was invited to the party but came without a costume). You decide how to share them out, and I'm not going to tell them that it was you who decided."
"Now, while you share them out, I have to review some papers. When you are finished distributing the bags, let me know, ok? I'll leave the three bags here for you."
When the participant has finished distributing the bags, he is asked:
23. "I see you have given the boy/girl without a costume the …". "Why?"
24. "You gave the boy/girl from the Robots the….". "Why?"
25. "You gave the boy/girl from the Pirates the…". "Why?"

Mixed story (Group and Intergroup)

First scene: "*Today, you and your schoolmates are visiting the zoo and you are all wearing yellow t-shirts. There, you have been invited to take part in a sack race against another visiting school, who are wearing blue t-shirts. The winning school will get a great prize. Only one boy/girl from each school will participate in the race.*"

Identification control questions:

1. "Do you like being from your school?";
2. "How much?";
3. "If you were told that you were now from the other school, would you like that?";
4. "How much?

Second scene: "*You and Danielle / Luke are the fastest, and both of you really want to participate to win the prize. But the class chooses Danielle / Luke to represent your school in the competition against another school.*"

***Emotional attribution questions*:**

5. *"How does Danielle / Luke feel when they are chosen to take part in the competition?"*
6. "*And you, how do you feel when they choose Danielle / Luke and not you?"*

Third scene: "*Betty/Christian is the fastest student from the other school. Both, Betty and Danielle [Christian and Luke] want to win the prize for their school.*"

Identification control question:

7. *"Who would you like to win?"*

Forth scene: *"Everything is ready for the sack race, Danielle / Luke is ready. When*

she/he leaves, she/he trips and falls to the ground, scratches her/his elbow and cannot continue, so Betty / Christian wins the race and the other school wins the prize."

***Emotional attribution questions*:**

8. *"How does Betty / Cristian feel when she/he sees that Danielle / Luke cannot continue the race?"*

9. *"And you, how do you feel, when you see that Danielle / Luke falls and cannot continue?*

10. "How good / bad?"

11. "Why?"

Allocation Task

"Do you remember what happened in this story? You were not chosen to compete in the sack race. They chose Danielle / Luke. At the start of the race, she/he fell down and the girl/boy from the other school won. So, the children from the other school won the award. "

Let's see what happens next. The zoo keepers gives you animal stickers. You have to share them out. "You are almost done. You have these three packages of stickers, and there are only three children left to get some stickers: Betty / Christian (the girl / boy from the other school), Danielle / Luke (who is in your group) and you. You decide how to share them out.

The experimenter shows participant the sticker packs (three different sizes) and three faces (the winner from the blue team, the loser from the yellow team, and the participant)

Before giving the sticker packages and in order for the participant not to feel pressured while deciding, the interviewer comments:

"While you share these three packages of stickers out, I have to read a message on my phone. When you are finished distributing them, let me know." The experimenter stands up and looks at his/her phone with his/her back to the child. When the child says that he/she has finished, the experimenter turns around and asks:

"Which one did you give Betty / Christian, the girl / boy from the other school?" "Why?"

"You have given Danielle / Luke, your schoolmate, the …." "Why?"

"And you have taken the ..." "Why did you take this package?"
