# Supplementary material for: Influence of Group Identification on Malicious and Benign Envy: A Cross-Sectional Developmental Study
Source: Front Psychol. 2021 Jun 30;12:663735. doi: 10.3389/fpsyg.2021.663735 (PMC8277992; doi:10.3389/fpsyg.2021.663735)
Supplement: Supplementary file 2 [file Table_1.pdf]

Table S1.

Frequencies and percentages of the combined arguments in the mixed category of justifications for the allocations by profile and experimental condition

Mixed Categories	INTERPERSONAL						GROUP					
	Malicious		Benign		Non Envious		Malicious		Benign		Non Envious	
Prosocial + Egocentric	3	75%	4	44%	4	21%	2	33%	2	20%	2	10%
Prosocial + Deservingness	0	0%	2	22%	15	79%	0	0%	5	50%	14	70%
Deservingness + Egocentric	1	25%	3	33%	0	0%	4	67%	3	30%	4	20%
Total	4	13%	9	28%	19	59%	6	16.7%	10	27.7%	20	55.6%

	INTERGROUP						MIXED					
	Malicious		Benign		Non Envious		Malicious		Benign		Non Envious	
Prosocial + Egocentric	1	12%	1	7%	3	10%	0	0%	0	0%	2	5%
Prosocial + Deservingness	0	0%	1	7%	19	63%	2	33%	3	23%	32	75%
Deservingness + Egocentric	2	25%	3	21.5%	1	3%	4	67%	2	15%	1	2%
Prosocial + In-group Fav.	2	25%	0	0%	5	17%	0	0%	0	0%	1	2%
Deservingness + In-group Fav.	3	38%	9	64.5%	2	7%	0	0%	2	15%	6	14%
In-group Fav. + Egocentric	0	0%	0	0%	0	0%	0	0%	6	47%	1	2%
Total	8	15%	14	27%	30	58%	6	10%	13	21%	43	69%
